# Relationship between tumor size and metastatic site in patients with stage IV non-small cell lung cancer: A large SEER-based study

**DOI:** 10.7717/peerj.7822

**Published:** 2019-10-10

**Authors:** Qinge Shan, Yanling Fan, Jun Guo, Xiao Han, Haiyong Wang, Zhehai Wang

**Affiliations:** 1 School of Medicine and Life Sciences, University of Jinan-Shandong Academy of Medical Sciences, Jinan, Shandong, China; 2 Department of Internal Medicine-Oncology, Shandong Cancer Hospital and Institute, Shandong First Medical University and Shandong Academy of Medical Sciences, Jinan, Shandong, China; 3 Department of Haematology and Oncology, Jinxiang People’s Hospital, Jinxiang Hospital Affiliated with Jining Medical University, Jining, Shandong, China

**Keywords:** Non-small cell lung cancer, Metastatic site, Metastasis, Tumor size, SEER

## Abstract

**Objective:**

To analyze the relationship between tumor size and metastatic site in stage IV NSCLC patients.

**Methods:**

A total of 40,196 stage IV NSCLC patients from 2010 to 2015 were screened by SEER database. Chi-square test was used to compare the characteristics of clinical variables. At the same time, multivariate Logistic regression analysis was used to evaluate the relationship between tumor size and organ metastasis.

**Results:**

Regardless of tumor size, the proportion of bone metastasis and lung metastasis was higher and similar in patients with squamous cell carcinoma, while in patients with adenocarcinoma, bone metastasis accounted for the highest proportion. We found that whether the metastatic site was bone, brain, liver or lung, the proportion of patients with a tumor size of 3–7 cm was the highest. Multivariate regression analysis demonstrated that patients with a tumor size of 3–7 cm and a tumor size ≥7 cm were more likely to develop brain metastasis and lung metastasis compared with patients with a tumor size ≤3 cm (all *P* < 0.001), which meant the larger the tumor, the greater the risk of brain or lung metastasis. At the same time, the results indicated that patients with a tumor size of 3–7 cm had a tendency to develop liver metastasis (*P* = 0.004), while the statistical significance was not found for patients with a tumor size ≥7 cm (*P* = 0.524). The results also revealed that patients with a tumor size of 3–7cm had no significant difference to develop bone metastasis (*P* = 0.116), while the statistical significance was found for patients with a tumor size ≥7 cm (*P* < 0.001).

**Conclusions:**

There was statistical significance between tumor size and metastatic site in patients with stage IV NSCLC. For brain or lung metastasis, the larger the tumor, the higher the risk of brain or lung metastasis. For liver metastasis, patients with a tumor size of 3–7 cm were more prone to develop liver metastasis. For bone metastasis, patients with a tumor size ≥7 cm were more likely to have bone metastasis.

## Introduction

Lung cancer is one of the most common malignancies and the leading cause of cancer death (Siegel, Miller & Jemal, 2018). The size, type and precise stage of the tumor determines the choice of treatment (Molina et al., 2008), which is beneficial for surgery, chemotherapy, radiotherapy and targeted therapy for the treatment of lung cancer (Cancer Council Australia Lung Cancer Guidelines Working Party, 2016). About 85% of lung cancer patients were diagnosed with non-small cell lung cancer (NSCLC), and approximately 40% were diagnosed with metastatic disease at the time of onset. The most common distant metastatic sites included the brain, liver, adrenal gland and bone (Govindan et al., 2006; Morgensztern et al., 2009; Lee et al., 2016). About 30% to 40% of NSCLC patients had bone metastasis during the course of their disease, and more than 60% of them had bone lesions at the initial diagnosis (Saad et al., 2007; Tsuya et al., 2007). Saad et al. (2008) showed that about 10% of NSCLC patients developed brain metastasis at the initial diagnosis, and 40–50% of NSCLC patients developed brain metastasis during the course of the disease. A retrospective study of 409 patients with stage IV non-small cell lung cancer showed that patients with liver metastasis had lower overall survival (OS) (Badawy et al., 2018).

Tumor size was a known prognostic factor for many cancers, including NSCLC. In most cases, larger tumors have poor prognosis (Motta et al., 1999). In a large clinical staging series (using the 6th edition TNM staging system), survival rates for patients with clinical T1 disease were significantly higher than those with T2 disease (HR 1.48, *P* < 0.0001). At the same time, the above study also demonstrated the survival of patients in clinical T1 group was significantly better than that in T 2-4 group (Shepherd et al., 2007).

Currently, few studies have focused on the relationship between tumor size and metastatic site in NSCLC patients. Therefore, this study retrospectively analyzed the correlation between tumor size and metastatic site in stage IV NSCLC patients.

## Materials & Methods

### Patient selection

The Surveillance Epidemiology and End Results (SEER) database was used to screened out appropriate patients with stage IV NSCLC according to the 7th TNM staging system (Surveillance, Epidemiology, and End Results , 2018). The SEER*Stat 8.3.5 software was used to screen stage IV NSCLC patients between 2010 and 2015. Patients included should meet the following criteria: microscopic confirmation; only one measurable primary tumor; and bone, brain, liver, or lung metastasis include at least one. The variables including age, race, sex, histology, tumor size, N stage, metastasis sites should have clear information. In addition, the histologic types of this study included only adenocarcinoma and squamous cell carcinoma.

### Ethical evaluation

Our study was approved by the ethics committee of the Shandong Cancer Hospital. Personally identifiable information was not involved therefore no informed consent was required.

### Statistical analysis

A chi-square test was used to compare the characteristics of clinical variables. At the same time, multivariate Logistic regression analysis was used to evaluate the relationship between tumor size and organ metastasis. All statistical analyses were conducted using SPSS 22.0 (SPSS, Chicago, IL, USA) software package. It was considered statistically significant when *p*-value ≤ 0.05 and all *p*-values were two-tailed.

## Results

### Patient demographics

According to study exclusion and inclusion criteria, a total of 40,196 stage IV NSCLC patients were enrolled. The cohort in our study included 18,197 (45.3%) female and 21,999 (54.7%) male patients, of whom 59.9% were over 65 years old. Most patients were of white racial background (76.9%, *n* = 30,894). Nearly three-quarters of the patients had a pathological type of adenocarcinoma (*n* = 29,269) and the rest were squamous cell carcinoma (*n* = 10,927). Patients with a tumor size of 3–7 cm accounted for more than half of the cohort (*n* = 21,266), followed by patients with a tumor size ≤3 cm (28.7%), and patients with a tumor size ≥7 cm (18.4%) at least, accounting for about one-fifth of the total. Among them, patients with bone metastasis, brain metastasis, liver metastasis and lung metastasis were 15,687 (39%), 11,048 (27.5%), 6,643 (16.5%), 12,760 (31.7%), respectively. Detailed patient data were summarized in Table 1.

**Table 1:** Characteristics of Patients from SEER Database according to different variables.

Variables	Number	%
**Age**		
<65	16,128	40.1
≥65	24,068	59.9
**Race**		
White	30,894	76.9
Black	5,477	13.6
Others	3,825	9.5
**Sex**		
Female	18,197	45.3
Male	21,999	54.7
**Histology**		
Adenocarcinoma	29,269	72.8
Squamous	10,927	27.2
**Tumor size**		
≤ 3 cm	11,541	28.7
3–7 cm	21,266	52.9
≥7 cm	7,389	18.4
**N stage**		
N0	9,737	24.2
N1	3,301	8.2
N2	18,565	46.2
N3	8,593	21.4
**Bone metastasis**		
Yes	15,687	39.0
No	24,509	61.0
**Brain metastasis**		
Yes	11,048	27.5
No	29,148	72.5
**Liver metastasis**		
Yes	6,643	16.5
No	33,553	83.5
**Lung metastasis**		
Yes	12,760	31.7
No	27,436	68.3

### Proportional distribution between tumor size and metastatic site

**Figure 1:**
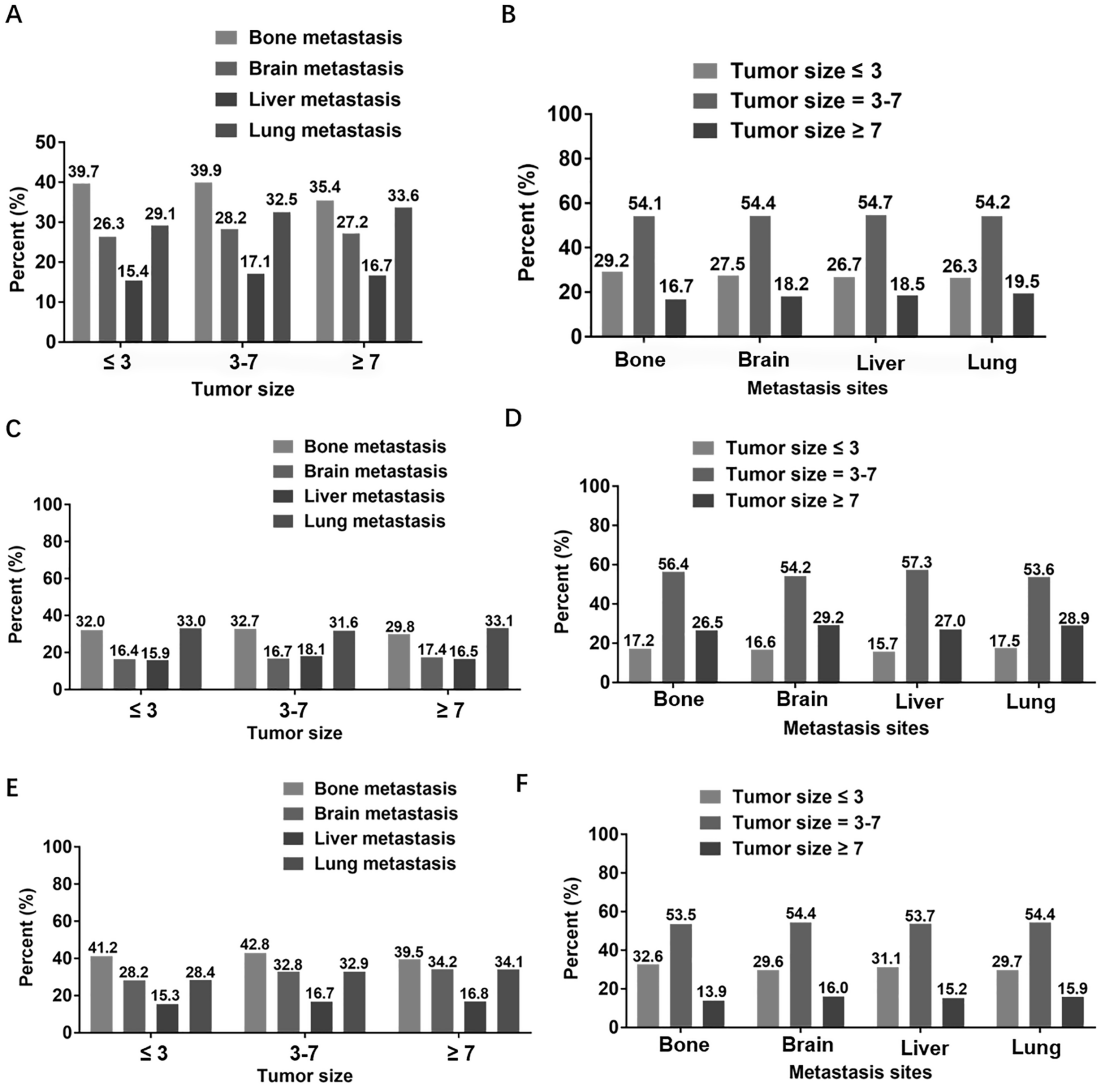
Proportional distribution between tumor size and metastasis sites. (A) In overall patients, the proportion of different metastasis sites under specific tumor size. (B) In overall patients, the proportion of different tumor sizes at specific metastasis site. (C) In patients with squamous cell carcinoma, the proportion of different metastasis sites under specific tumor size. (D) In patients with squamous cell carcinoma, the proportion of different tumor sizes at specific metastasis site. (E) In patients with adenocarcinoma, the proportion of different metastasis sites under specific tumor size. (F) In patients with adenocarcinoma, the proportion of different tumor sizes at specific metastasis site.

The Fig. 1 showed the proportion of different metastatic sites in patients with a particular tumor size, as well as the proportion of different tumor sizes in patients with specific metastatic site. Firstly, we analyzed the percentage of different metastatic sites in patients with fixed tumor size in all patients (Fig. 1A). It could be seen from the histogram that, regardless of the size of the tumor, the number of overall patients with bone metastasis was the largest, followed by lung metastasis and brain metastasis, and liver metastasis was the least. Secondly, we analyzed the subgroup of two different pathological types of NSCLC patients. For patients with squamous cell carcinoma (Fig. 1C), regardless of tumor size, the higher proportion was bone metastasis and lung metastasis, and the proportion was similar, while the lower proportion was brain metastasis and liver metastasis, and the proportion was almost equal. For patients with adenocarcinoma (Fig. 1E), regardless of the size of the tumor, the highest proportion was in patients with bone metastasis and the least in patients with liver metastasis. In addition, we exchanged horizontal and vertical coordinates for the same analysis. We first analyzed the proportion of different tumor sizes in patients with specific metastatic site in all patients. We could find that whether the metastatic site was bone, brain, liver or lung, the proportion of overall patients with a tumor size of 3–7 cm was the highest, accounting for more than half, and the proportion of patients with a tumor size ≥7 cm was the lowest, accounting for less than 20% (Fig. 1B). Regardless of the metastasis site, the proportion of tumor size of 3 to 7 cm was the highest and that of tumor size ≤3 cm was the lowest in patients with squamous cell carcinoma (Fig. 1D). Similarly, regardless of the metastasis site, patients with a tumor size of 3–7 cm accounted for the highest proportion of patients with adenocarcinoma, and the lowest proportion was ≥7 cm (Fig. 1F).

### Relationship between tumor size and metastatic site

To further explore the relationship between tumor size and metastatic sites, we performed a multivariate regression analysis (Table 2). The results showed that patients with a tumor size of 3–7 cm and a tumor size ≥7 cm were more likely to develop brain metastasis and lung metastasis compared with patients with a tumor size ≤3 cm (all *P* < 0.001), which meant the larger the tumor, the greater the risk of brain or lung metastasis. Interestingly, compared with patients with a tumor size ≤3 cm, patients with a tumor size of 3–7 cm had a tendency to develop liver metastasis (*P* = 0.004), while the statistical significance was not found for patients with a tumor size ≥7 cm (*P* = 0.524). In addition, compared with patients with a tumor size ≤3 cm, patients with a tumor size of 3–7 cm had no significant difference to develop bone metastasis (*P* = 0.116), while the statistical significance was found for patients with a tumor size ≥7 cm (*P* < 0.001).

**Table 2:** Multivariate Logistic regression analyses to analyze the relationship between different variables and metastatic site.

Variables	Bone metastasis		Brain metastasis		Liver metastasis		Lung metastasis	
	HR (95% CI)	*P*	HR (95% CI)	*P*	HR (95% CI)	*P*	HR (95% CI)	*P*
**Age**		<0.001		<0.001		0.018		<0.001
<65	Reference		Reference		Reference		Reference	
≥65	0.887 (0.850–0.924)	<0.001	0.552 (0.527–0.578)	<0.001	0.936 (0.887–0.989)	0.018	1.232 (1.179–1.287)	<0.001
**Race**		<0.001		<0.001		0.020		<0.001
White	Reference		Reference		Reference		Reference	
Black	0.851 (0.801–0.904)	<0.001	0.867 (0.811–0.928)	0.057	0.892 (0.824–0.966)	0.005	1.075 (1.010–1.144)	0.024
Others	1.041 (0.971–1.115)	0.259	1.079 (1.001–1.162)	0.390	0.983 (0.897–1.077)	0.710	1.234 (1.149–1.325)	<0.001
**Sex**		<0.001		<0.001		0.001		<0.001
Female	Reference		Reference		Reference		Reference	
Male	1.226 (1.177–1.278)	<0.001	0.889 (0.850–0.931)	<0.001	1.093 (1.036–1.153)	0.001	0.918 (0.879–0.958)	<0.001
**Histology**		<0.001		<0.001		0.068		0.464
Adenocarcinoma	Reference		Reference		Reference		Reference	
Squamous	0.650 (0.619–0.682)	<0.001	0.457 (0.431–0.484)	<0.001	1.058 (0.996–1.124)	0.068	1.019 (0.970–1.070)	0.464
**Tumor Size**		<0.001		<0.001		0.009		<0.001
≤ 3 cm	Reference		Reference		Reference		Reference	
3–7 cm	1.038 (0.991–1.089)	0.116	1.221 (1.158–1.286)	<0.001	1.097 (1.030–1.167)	0.004	1.158 (1.102–1.218)	<0.001
≥7 cm	0.877 (0.824–0.934)	<0.001	1.271 (1.186–1.361)	<0.001	1.027 (0.947–1.114)	0.524	1.207 (1.132–1.286)	<0.001
**N stage**		<0.001		<0.001		<0.001		<0.001
N0	Reference		Reference		Reference		Reference	
N1	1.297 (1.195–1.408)	<0.001	1.169 (1.069–1.278)	0.001	1.304 (1.110–1.532)	0.001	0.959 (0.877–1.049)	0.361
N2	1.332 (1.264–1.402)	<0.001	1.074 (1.014–1.137)	0.015	1.510 (1.362–1.673)	<0.001	1.202 (1.138–1.270)	<0.001
N3	1.306 (1.229–1.388)	<0.001	0.961 (0.899–1.029)	0.254	1.473 (1.304–1.664)	<0.001	1.869 (1.755–1.989)	<0.001

## Discussion

The International Association for the Study of Lung Cancer (IASLC) revised and released the eighth TNM staging system in 2015 (Detterbeck et al., 2016a; Detterbeck et al., 2016b; Detterbeck et al., 2016c; Detterbeck et al., 2016d; Kim, 2016; Travis et al., 2016). According to the 8th TNM staging system, T1 was defined as tumor size ≤3 cm, T2 was defined as tumor size of 3–5 cm (including upper limit), T3 was defined as tumor size of 5–7 cm (including upper limit), and T4 was defined as tumor size >7 cm. In the 7th edition of TNM staging, the tumor size ≤3 cm was T1, and the tumor size greater than 7 cm was T3. In our study, we divided into three groups according to tumor size, namely tumor size ≤3 cm, ≥7 cm and tumor size of 3–7 cm. Our study indicated that patients with a tumor size of 3–7 cm had a tendency to have brain metastasis, liver metastasis and lung metastasis. Meanwhile, the results also demonstrated that patients with a tumor size ≥7 cm were more prone to have bone metastasis, brain metastasis, lung metastasis.

Many retrospective studies had shown that tumor size was an important prognostic factor for NSCLC patients (Basaki et al., 2006; Bradley et al., 2002; Dehing-Oberije et al., 2008; Etiz et al., 2002; Stinchcombe et al., 2006). This corresponded to the hypothesis that larger tumors might produce more clonogenic cells, at the same time, it was associated with clinical observation of tumor size and local therapy (Dubben, Thames & Beck-Bornholdt, 1998). Zhang et al. (2015) showed that in patients with early or locally advanced stage NSCLC, as well as in patients with lymph node positive, larger primary tumors were associated with poorer prognosis. In addition, Yang et al. (2010) selected 917 patients who underwent surgery for retrospective analysis, and found that the smaller the tumor, the higher the proportion of N0M0, indicating that the smaller the tumor size, the earlier the staging. A study (Ball et al., 2013) involving 868 NSCLC patients showed that the survival rate of patients with a tumor size ≤3 cm was significantly higher than that of patients with a tumor size of 3–7 cm (including upper limit). Interestingly, there was no statistically significant difference between patients with a tumor size >7 cm and those with tumors greater than 5 cm and less than or equal to 7 cm (*P* = 0.11), although the former had the highest survival rate (Ball et al., 2013). While, our study drew the conclusion that in patients with brain or lung metastasis, the larger the tumor, the greater the risk of brain or lung metastasis. To a certain extent, it could be considered that different tumor sizes may cause different prognosis. Jin, Chen & Yu (2016) revealed that lymph node involvement in large tumors is higher than in small tumors: the incidence of lymph node metastasis was zero in patients with lesions <1 cm, 20% in lesions of 1–2 cm, 37.6% in lesions of 2–3 cm, and 66.7% in lesions >7 cm, which supported the theory that tumor size could embody malignant behavior.

A previous study had reported that liver infiltration was a poor prognostic signal and patients with liver metastasis had significantly lower survival expectations than those with other metastatic diseases (Hoang et al., 2012). In addition, subjects with liver involvement received less clinical benefit from chemotherapy (Castañón et al., 2015). Our study showed no statistically significant difference in the risk of liver metastasis in patients with large tumors. Therefore, we could speculate that small tumors might be controlled by local treatment. When the tumor was large, the survival period of the patients was too short to obtain the correlation between tumor size and the risk of liver metastasis due to the limitations of treatment. Previous studies suggested that patients with brain metastases had a good prognosis, probably due to whole brain irradiation in patients with brain metastasis (Gray et al., 2014; Maclean et al., 2013). A univariate analysis displayed that pulmonary metastasis was not an independent prognostic factor in NSCLC patients (Tamura et al., 2015). Homogeneous risk factors for morbidity and survival outcomes in NSCLC patients with bone metastasis included male (OR 0.815, 95% CI [0.771–0.861], *P* < 0.001) and higher T-stage (OR 1.287, 95% CI [1.251–1.324], *P* < 0.001). In addition, these clinical factors were able to be combined with biomarkers to more accurately predict bone metastasis in NSCLC patients, contributing to the early diagnosis and early intervention of bone metastasis (Song et al., 2019).

Lung cancer was a heterogeneous disease, with different subtypes showing different genetic variation. Also, different subtypes also had their own specific variants, such as MET in adenocarcinoma, FGFR1 and FGFR3 in squamous cell carcinoma and MYC in small cell lung cancer (Zhang et al., 2017). This might explain the different proportion of metastatic sites in different NSCLC subtypes with the same tumor size in this study. Secondly, the abnormality of epigenetics would lead to intratumoral heterogeneity, which could also be the cause of the above results (Dong et al., 2017). In addition, Xu et al. (2017) found that the expression of different kinds of mucins in lung cancer subtypes led to inter-tumor heterogeneity, which could also be one of the reasons. Unfortunately, due to the lack of access to such information in the database, this study had not carried out the analysis of tumor biology, which needed to be verified and improved by prospective researches.

The advantage of this study was that the sample size was relatively large, and by extracting data from the SEER database, the relationship between tumor size and metastasis sites in stage IV NSCLC patients was well analyzed. Inevitably, there were some limitations in our study. Firstly, as a retrospective study, although the sample size was relatively large, we could not avoid selection bias. Secondly, other variables, such as smoking history, mutation status, performance status, and treatment strategies that might affect prognosis were not included in this study. In addition, we did not collect information for other metastatic sites, such as adrenal glands, which might lead to underestimation of other metastatic sites. Therefore, a series of prospective studies are needed to verify these conclusions.

## Conclusions

There was statistical significance between tumor size and metastatic site in patients with stage IV NSCLC. For brain or lung metastasis, the larger the tumor, the more likely it was to develop metastasis. For liver metastasis, patients with a tumor size of 3–7 cm were more prone to develop liver metastasis. For bone metastasis, patients with a tumor size ≥7 cm were more likely to have bone metastasis.

##  Supplemental Information

10.7717/peerj.7822/supp-1 Data S1 Raw Data

## Additional Information and Declarations

### Data Availability

The following information was supplied regarding data availability: The raw measurements are available in Data S1.
